# Reduction of Interhemispheric Homotopic Connectivity in Cognitive and Visual Information Processing Pathways in Patients With Thyroid-Associated Ophthalmopathy

**DOI:** 10.3389/fnhum.2022.882114

**Published:** 2022-06-30

**Authors:** Chen-Xing Qi, Zhi Wen, Xin Huang

**Affiliations:** ^1^School of Optometry and Ophthalmology and Eye Hospital, Wenzhou Medical University, Wenzhou, China; ^2^Department of Radiology, Renmin Hospital of Wuhan University, Wuhan, China; ^3^Department of Ophthalmology, Jiangxi Provincial People's Hospital, The First Affiliated Hospital of Nanchang Medical College, Nanchang University, Nanchang, China

**Keywords:** thyroid-associated ophthalmopathy, functional magnetic resonance imaging, voxel-mirrored homotopic connectivity, functional connectivity, support vector machine

## Abstract

**Purpose:**

Thyroid-associated ophthalmopathy (TAO) is a vision threatening autoimmune and inflammatory orbital disease, and has been reported to be associated with a wide range of structural and functional abnormalities of bilateral hemispheres. However, whether the interhemisphere functional connectivity (FC) of TAO patients is altered still remain unclear. A new technique called voxel-mirrored homotopic connectivity (VMHC) combined with support vector machine (SVM) method was used in the present study to explore interhemispheric homotopic functional connectivity alterations in patients with TAO.

**Methods:**

A total of 21 TAO patients (14 males and 7 females) and 21 wellmatched healthy controls (HCs, 14 males and 7 females), respectively, underwent functional magnetic resonance imaging (fMRI) scanning in the resting state. We evaluated alterations in the resting state functional connectivity between hemispheres by applying VMHC method and then selected these abnormal brain regions as seed areas for subsequent study using FC method. Furthermore, the observed changes of regions in the VMHC analysis were chosen as classification features to differentiate patients with TAO from HCs through support vector machine (SVM) method.

**Results:**

The results showed that compared with HCs, TAO patients showed significantly lower VMHC values in the bilateral postcentral gyrus, lingual gyrus, calcarine, middle temporal gyrus, middle occipital gyrus and angular. Moreover, significantly decreased FC values were found between the right postcentral gyrus/lingual gyrus/calcarine and left lingual gyrus/cuneus/superior occipital gyrus, left postcentral gyrus/lingual gyrus/calcarine and right lingual gyrus/ middle temporal gyrus, right middle temporal gyrus and left cerebellum-8/lingual gyrus/middle occipital gyrus/supplementary motor area, left middle temporal gyrus and right middle occipital gyrus, right middle occipital gyrus/angular and left middle temporal pole (voxel-level *p* < 0.01, Gaussian random field correction, cluster-level *p* < 0.05). The SVM classification model achieved good performance in differentiating TAO patients from HCs (total accuracy: 73.81%; area under the curve: 0.79).

**Conclusion:**

The present study revealed that the altered interhemisphere interaction and integration of information involved in cognitive and visual information processing pathways including the postcentral gyrus, cuneus, cerebellum, angular, widespread visual cortex and temporal cortex in patients with TAO relative to HC group. VMHC variability had potential value for accurately and specifically distinguishing patients with TAO from HCs. The new findings may provide novel insights into the neurological mechanisms underlying visual and cognitive disorders in patients with TAO.

## Introduction

Hyperthyroidism occurs mainly in adults between the ages of 20 and 40 years, with an incidence of ~2% (Reddy et al., 2017). In these patients, thyroid hormone levels inside and outside of the thyroid gland increase for various reasons. Thyroid hormone enters the blood circulation and has systemic effects on tissues and organs, resulting in broadly increased excitability and hypermetabolism (Tu et al., 2020). The most common extrathyroidal complication of hyperthyroidism is thyroid-associated ophthalmopathy (TAO). The clinical course of this disease is generally divided into acute inflammatory and chronic quiescent stages. The most common physical complaints in patients with TAO are exophthalmos, eyelid swelling, lid retraction and impaired visual function (Du et al., 2021). Lesions in the visual pathway are mainly caused by periorbital edema that compresses the optic nerve, retinal ganglion cell damage because of abnormal thyroid hormone levels, and high intraocular pressure (Zah-Bi et al., 2019). TAO is a multifactorial inflammatory and autoimmune orbital disease; thus, affected patients exhibit ocular symptoms, along with affective dysfunction and cognitive deficits (Coulter et al., 2007).

In recent years, the development of functional neuroimaging techniques has enabled the identification of visual and cognitive dysfunction in patients with TAO; these are associated with local structural and functional abnormalities in the corresponding brain regions. For example, a study using diffusion tensor imaging technique found that patients with TAO had significant microstructural changes in optic nerve (Liu et al., 2022). Investigations of neural metabolic activity in the brains of TAO patients showed abnormal metabolic activities in the prefrontal cortex, which were closely associated with poor performance on neuropsychological tests (Bhatara et al., 1998). Wu et al. (2020) reported that patients with TAO exhibited gray matter loss and lower fractional anisotropy in brain regions corresponding to visual and cognitive dysfunction. Graph theoretical analysis showed that structural brain connectome disruption was present in patients with TAO, in relation to psychiatric dysfunction (Wu et al., 2021). In addition to showing structural changes in the brain, patients with TAO have also been reported to have functional disturbances in brain regions. A regional homogeneity algorithm revealed that hyperthyroidism patients with dysthyroid optic neuropathy had abnormal neural activity in the limbic system (Jiang et al., 2021). Using functional magnetic resonance imaging (fMRI) method, Chen et al. (2021b) found aberrant spontaneous brain activities in regions associated with cognitive and visual deficits in patients with TAO; these findings indicated that careful monitoring is needed for neuropsychological disturbances in such patients during diagnosis and treatment. Thus far, research has consistently shown that patients with TAO have structural and neural function alterations in vision-related and cognition-related brain regions. Nevertheless, it remains unclear whether an abnormal functional interaction exists between the two cerebral hemispheres in patients with TAO.

Research by Foubert et al. (2010) showed that synchronization between cerebral hemispheres is closely associated with the visual experiences. Interhemispheric homotopic connectivity has been proposed as a basic principle of functional architecture that integrates brain functions underlying coherent cognition, visual processing, and consciousness (Zuo et al., 2010). Resting-state fMRI constitutes a noninvasive and repeatable method to directly quantify interhemispheric functional connectivities (FCs). Originating from resting brain symmetrical voxels, voxel-mirrored homotopic connectivity (VMHC) is an index that can be used to measure the synchronization between two hemispheres. The strength of functional connection between a voxel and its corresponding voxels in the contralateral hemisphere is calculated using the VMHC algorithm. Abnormal connections involving multiple symmetrical regions in the two hemispheres can reflect coordinated neuronal activity in the right and left hemispheres. Because it enables characterization of the intrinsic functional architecture of the brain, the VMHC algorithm has recently been used to investigate brain function in common ocular diseases (Foubert et al., 2010; Liang et al., 2017). Seed-based FC in rs-fMRI is a measure of the spatiotemporal synchrony or correlations of the blood oxygenation level-dependent (BOLD) signal among anatomically distinct brain regions. VMHC can only reflect changes in functional connectivity in both hemispheres. The seed-based FC method reflect the region of interests (ROI) to whole brain FC, which indicate the whole brain network communication. In order to comprehensively analyze the changes of brain function in TAO patients, we combined VMHC technology and FC technology in this study.

Machine learning (ML), the core algorithm of artificial intelligence, has provided a systematic approach for using a set of high-dimensional data to make predictions. Furthermore, ML is a promising method in ophthalmology research because of its precision, non-invasiveness, and repeatability; it can be used for the diagnosis of ocular diseases and for the evaluation of therapeutic effects in those diseases. Support vector machine (SVM) is a supervised learning algorithm with a unique ability to classify disease-state and disease-free conditions; it has great potential for finding subtle differences in spatial patterns of structural and functional brain images. Tong et al. (2021) assessed the combined effects of an SVM model and FC analysis to classify iridocyclitis patients and healthy controls (HCs), with a substantial accuracy of 67.31%. Cui et al. (2020) also reported that the SVM approach provided robust accuracy in the classification of patients with generalized anxiety disorder through evaluations of the dynamic alterations of amplitude of low-frequency fluctuations metrics. To our knowledge, this was the first study that applied the SVM technique to VMHC data for predictive investigations in patients with TAO.

Here, we investigated changes in interhemispheric FC among patients with TAO by means of VMHC metrics. Subsequently, we used a seed-based FC method to detect potential alterations in interregional connectivity between regions with abnormal VMHC values and other brain areas. We then used the observed alterations in the VMHC algorithm as classification features to examine whether these alterations could provide substantial accuracy and specificity for differentiating patients with TAO from HCs *via* SVM classification.

## Methods

### Subjects

The work recruited a total of 21 right-handed patients with TAO (14 males and 7 females, mean age: 54.17 ± 4.83) from ophthalmology and endocrinology departments of Jiangxi Provincial People's Hospital. The inclusion criteria of TAO patients into experimental group included the following details: (1) these patients were all in the acute inflammatory stage of TAO with vision loss and definite ocular symptoms (i.e., clear evidence of impaired visual function, swelling of eyelids, lid retraction and exophthalmos); (2) ability to complete the fMRI scanning (no cardiac pacemaker, implanted metal devices or insulin pump); (3) no history or clinical evidence of any other diseases including cardiovascular diseases, neurological diseases, psychiatric disorders, brain parenchyma diseases and systematic diseases. Subjects were excluded in the research if they presented as one of the following conditions: (1) other ocular diseases besides TAO (glaucoma, vitreous hemorrhage, high myopia, strabismus, etc.); (2) previous history of ocular surgery and eye trauma; (3) breastfeeding and pregnant women.

During the same period, 21 HCs (14 males and 7 females, mean age: 55.17 ± 5.37) matched with similar age, sex, handedness and academic years were recruited to participate the study. And the subjects must presented with no eye disease, no brain neurological disease or systematic disease, no alcohol or drug addiction and no contraindications for fMRI scanning—details of the study participants were the same as our previous work (Qi et al., 2021).

This cross-sectional, retrospective study was approved by the institutional review board of Jiangxi Provincial People's Hospital and all research methods strictly adhered to the Declaration of Helsinki. Participants enrolled in the study of their own accord and were informed of the purpose, methods, as well as potential risks before signing an informed consent form.

### MRI Data Acquisition

MRI scanning was performed on a 3-Tesla MR scanner (GE Healthcare, Milwaukee, WI, USA) with an eight-channel head coil. All participants were required to close their eyes without falling asleep when undergoing MRI scanning. The T1 parameters (repetition time = 8.5 ms, echo time = 3.3 ms, thickness = 1.0 mm, gap = 0 mm, acquisition matrix = 256 × 256, field of view = 240 × 240 mm^2^ and flip angle = 12°) and 240 functional images parameters (repetition time = 2000 ms, echo time = 25 ms, thickness = 3.0 mm, gap = 1.2 mm, acquisition matrix = 64 × 64, field of view = 240 × 240 mm^2^, flip angle = 90°, voxel size = 3.6 × 3.6 × 3.6 mm^3^ and 35 axial slices) covering the whole brain for all participants were obtained.

### FMRI Data Pre-processing

All preprocessing was performed using the toolbox for Data Processing & Analysis of Brain Imaging–Version4.1 (DPABI, http://www.rfmri.org/dpabi), which is based on Statistical Parametric Mapping (SPM8) (http://www.fil.ion.ucl.ac.uk) implemented in MATLAB 2013a (MathWorks, Natick, MA, USA) and briefly following the steps: (1) The first 10 volumes of each subject were removed for the signal reaching equilibrium. (2) Slice timing and head motion correction were conducted. For head motion parameters, more than 2 mm in any direction of x, y, and z or for whom rotation exceeded 2° of any angle during scanning were excluded. (3) Individual 3D-BRAVO images were registered to the mean fMRI data, then resulting aligned T1-weighted images were segmented using the Diffeomorphic Anatomical Registration Through Exponentiated Lie Algebra (DARTEL) approach normalized to the Montreal Neurological Institute (MNI) space. 4) Linear regression analysis was applied to regress out several covariates (Friston-24 head motion parameters, global brain signal, and averaged signal from cerebrospinal fluid and white matter). 5) The fMRI data with linear trend were removed, and temporal band-pass was filtered (0.01–0.08 Hz) to reduce the influence of noise.

### VMHC Analysis

To evaluate the interhemispheric connectivity, VMHC method was performed using the DPABI toolkit according to a previous research (Zuo et al., 2010). Briefly, T1 structural images of all participants were normalized to generate a symmetrical template of left and right hemispheres to reduce the geometric difference between brain hemispheres. Then, the same transformation of the T1 images was performed on the preprocessed functional images.

The VMHC values was calculated each pair of symmetrical interhemispheric voxel's time series (Pearson's correlation) at each voxel as interhemispheric resting-state homotopic connectivity (VMHC). Moreover, this symmetric T1 template did not include the brain midline (X = 0) area. Finally, the generated correlation values were performed with Fisher' z transformation to obtain the zVMHC maps for further analysis and help to improve normality.

### Seed-Based FC Analysis

The DPABI toolkit was also applied to define the regions of interest (ROI) so as to investigate whether abnormal FC existed between the brain regions with altered VMHC values and other brain regions. We defined the center of regions with altered VMHC as the seed point and set the radius of the sphere ROI as 3 mm to compute FC signal values. For each participant, we first averaged the time series of voxels within each ROI, and next computed the Pearson's correlation coefficient for the subjects between the representative time series of the ROIs and the time series of other cerebrum regions. Finally, to minimize the influence of individual differences on statistical comparison, we applied Fisher' z transformation for converting the generated correlations-coefficient maps to zFC values.

### Statistical Analysis

The cumulative clinical measurements were analyzed in this study using SPSS 20.0 (SPSS Inc, Chicago, IL, USA). Chi-square test was adopted to evaluate categorical variables, while Independent two-sample t-test was applied to analyse continuous variables. A *p* value < 0.05 was considered statistically significant.

A two-sample t-test was used to investigate the difference in the zVMHC maps and z-value FC maps between the two groups with DPABI toolkit after controlling for the effects of age and sex. Gaussian random field (GRF) method was used to correct for multiple comparisions of VMHC and FC differences between the two groups (two-tailed, voxel-level *p* < 0.01, GRF correction, cluster-level *p* < 0.05).

### SVM Analysis

To evaluate whether the VMHC metrics alterations could serve as potential diagnostic metrics for TAO, we performed ML analyses using SVM algorithm with the average VMHC values of all clusters showing significant intragroup differences as the features.

Mapping nonlinear data to a high dimension feature space and finding a linear separating hyperplane to separate the two-group data are the core idea of SVM algorithm. In this study, we used the Gaussian radial basis function kernel SVM, implement in the LIBSVM software package (http://www.csie.ntu.edu.tw/~cjlin/libsvm/), to investigate the potential diagnostic of dynamic metrics. The C in the SVM was set to 1 and radial basis function kernel parameter γ was optimized among the values of 2N (N from 4 to 4), and a leave-one-out cross-validation (LOOCV) was applied to validate the performance of our proposed approach. It involved excluding a participant from each group for test and training the classifier using the remaining participants. This procedure was repeated for each participant to assess the overall accuracy of the SVM. To quantify the performance of classification methods, accuracy, sensitivity and specificity were reported. Besides the classification accuracy, the receiver operating characteristic (ROC) curves and the corresponding area under the curve (AUC) were also computed to evaluate the classification efficiency.

## Results

### Demographics and Disease Characteristics

There were no statistically significant differences between the TAO and HC groups in gender (*P* > 0.999) or age (*P* = 0.745), but significant differences in best corrected visual acuity of left eye (*p* = 0.023), right eye (*p* = 0.026), and intraocular pressure (*p* < 0.001). The results of these clinical data were summarized in Table 1.

**Table 1:** Characteristics of participants included in the study.

**Condition**	**TAO group**	**HC group**	***t***	*P*-value*
Gender (male/female)	14/7	14/7	N/A	>0.999
Age (years)	54.17 ± 4.83	55.17 ± 5.37	−0.348	0.745
Duration (months)	11.25 ± 4.42	N/A	N/A	N/A
BCVA-OD	0.67 ± 0.35	1.14 ± 0.15	−4.462	0.026
BCVA-OS	0.64 ± 0.29	1.06 ± 0.23	−4.297	0.023
IOP	25.63 ± 3.36	14.45 ± 1.07	5.532	<0.001
Education	11.17 ± 2.64	11.42 ± 1.95	−0.269	0.861

*Independent samples t-test for the other normally distributed continuous data (means ± SD).*

* *p < 0.05 indicated significant differences.*

*TAO, thyroid-associated ophthalmopathy; HC, healthy control; N/A, not applicable; BCVA, best corrected visual acuity; OD, oculus dexter; OS, oculus sinister; IOP, intraocular pressure*.

### Comparisons of VMHC Between Patients With TAO and HCs

**Figure 1:**
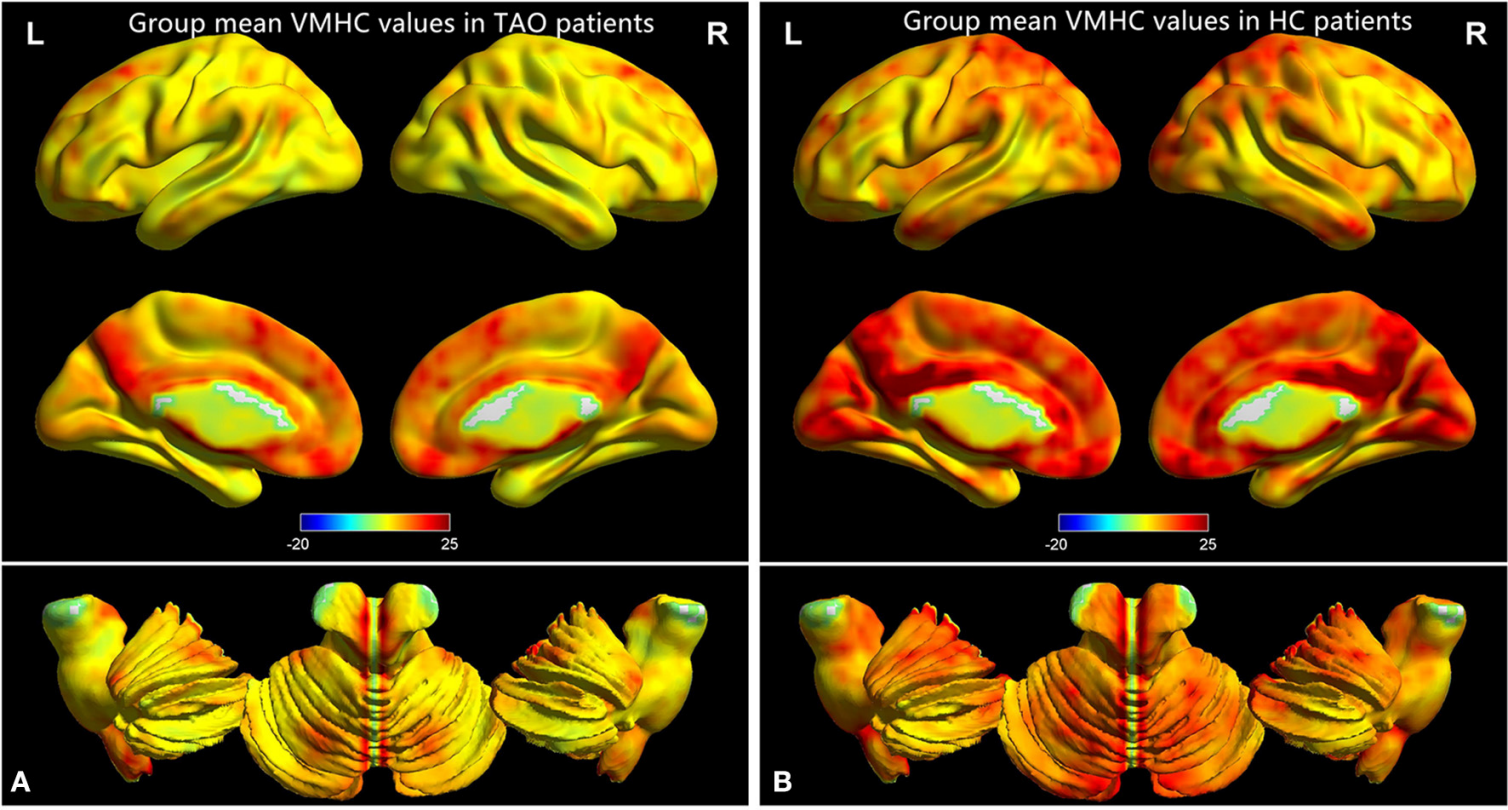
One-sample t-test results of VMHC maps within TAO group **(A)** and HC group **(B)**. TAO, thyroid-associated ophthalmopathy; VMHC, voxel-mirrored homotopic connectivity; HC, healthy control.

**Figure 2:**
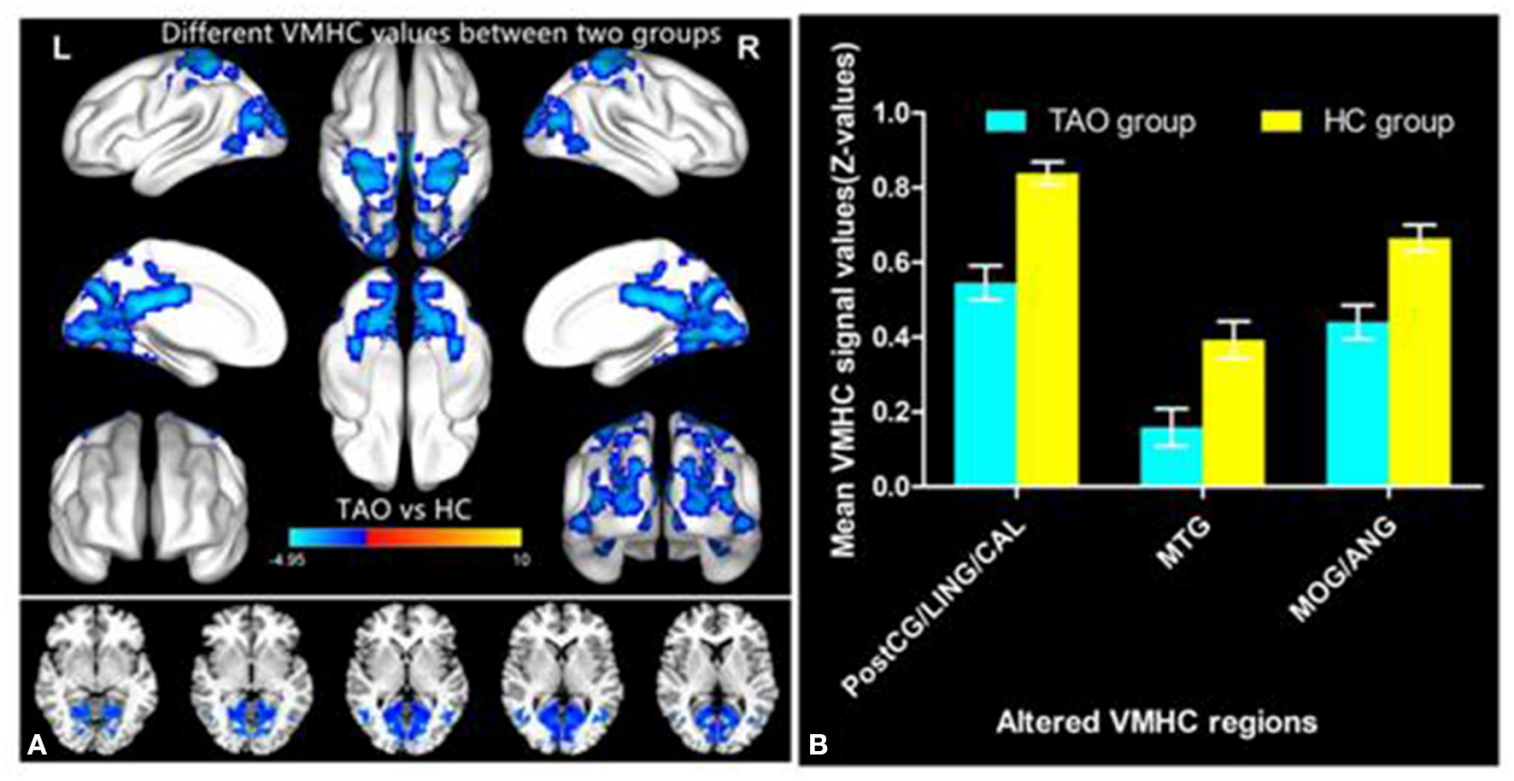
Significant VMHC differences in the TAO and HC group **(A)**; The mean of altered VMHC values between patients with TAO and HCs **(B)**. TAO, thyroid-associated ophthalmopathy; HC, healthy control; VMHC, voxel-mirrored homotopic connectivity; PostCG, postcentral gyrus; LING, lingual gyrus; CAL, calcarine; MTG, middle temporal gyrus; MOG, middle occipital gyrus; ANG, angular gyrus.

Figure 1 showed the spatial distribution of global VMHC maps of the TAO and HCs within each group. Compared with the HCs, patients with TAO showed significantly lower VMHC values in the bilateral postcentral gyrus (PostCG), lingual gyrus (LING), calcarine (CAL), middle temporal gyrus (MTG), middle occipital gyrus (MOG) and angular (ANG) (voxel-level *p* < 0.01, GRF correction, cluster-level *p* < 0.05). Detailed information for these brain areas with altered VMHC values between the two groups was shown in Table 2 and Figure 2.

**Table 2:** Brain areas with significantly altered VMHC values between two groups.

**Regions**	**Cluster** **size**	**Brodmann's** **areas**	**MNI coordination**			***T* value**
			**X**	**Y**	**Z**	
R/L-PostCG/LING/CAL	2,243	3/4/18/19	± 3	−21	27	−4.9477
R/L-MTG	13	-	± 39	−63	3	−3.0776
R/L-MOG/ANG	27	19/39	± 45	−78	33	−3.3491

*TAO, thyroid-associated ophthalmopathy; MNI, Montreal Neurological Institute; VMHC, voxel-mirrored homotopic connectivity; PostCG, postcentral gyrus; LING, lingual gyrus; CAL, calcarine; MTG, middle temporal gyrus; MOG, middle occipital gyrus; ANG, angular gyrus*.

### Differences in FC

**Figure 3:**
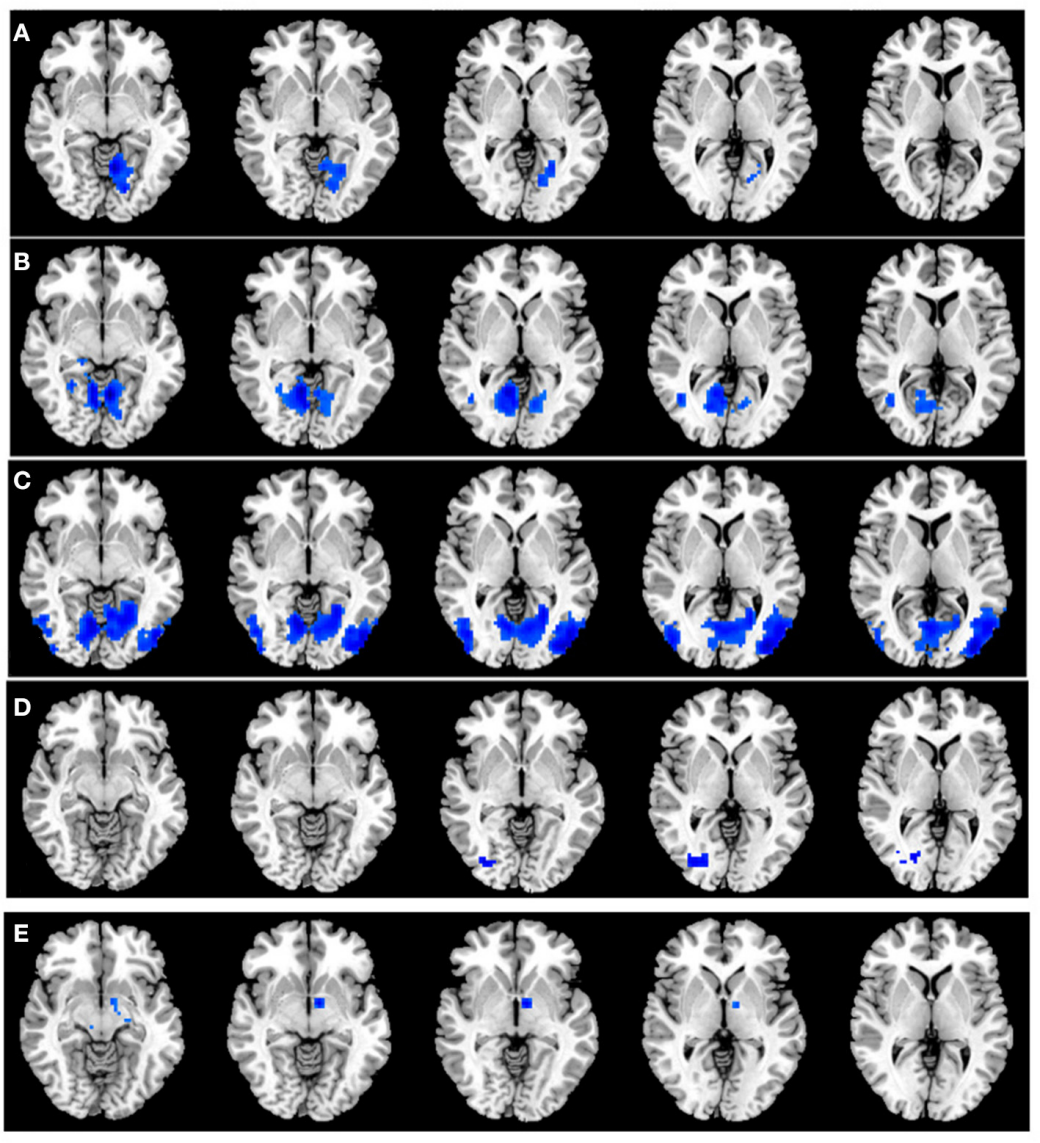
**(A)** Significant FC differences between the right PostCG/LING/CAL and left LING/CUN/SOG. **(B)** Significant FC differences between the left PostCG/LING/CAL and right LING/MTG. **(C)** Significant FC differences between the right MTG and left CER-8/LING/MOG/SMA. **(D)** Significant FC differences between the left MTG and right MOG. **(E)** Significant FC differences between the right MOG/ANG and left MTP. TAO, thyroid-associated ophthalmopathy; FC, functional connectivity; PostCG, postcentral gyrus; LING, lingual gyrus; CAL, calcarine; MTG, middle temporal gyrus; MOG, middle occipital gyrus; ANG, angular gyrus; CUN, cuneus; SOG, superior occipital gyrus; CER, cerebellum; SMA, supplementary motor area; MTP, middle temporal pole.

A total of six seed ROIs (three per hemisphere) were applied for further FC analysis, which were derived from those regions showing significant differences in the VMHC values. We finally found TAO patients exhibited decreased FC values between the right PostCG/LING/CAL and left LING/cuneus (CUN)/superior occipital gyrus(SOG), left PostCG/LING/CAL and right LING/MTG, right MTG and left cerebellum-8 (CER-8)/LING/MOG/supplementary motor area (SMA), left MTG and right MOG, right MOG/ANG and left middle temporal pole (MTP) (voxel-level *p* < 0.01, GRF correction, cluster-level *p* < 0.05), which were illustrated in Table 3 and Figure 3. But the left MOG/ANG of TAO patients didn't shown significantly higher or lower FC values with other brain regions than that of HCs.

**Table 3:** Significant differences in seed-based FC between TAO patients and HCs.

**Seed region**	**Regions**	**Cluster** **size**	**MNI coordination**			***P* value**
			**X**	**Y**	**Z**	
R-PostCG/LING/CAL	L-LING	282	−12	−60	−12	−4.5011
	L-CUN	163	3	−93	24	−3.9564
	L-SOG	90	−24	−72	18	−3.8977
L-PostCG/LING/CAL	R-LING	852	−6	−60	−6	−4.7800
	R-MTG	29	42	−63	3	−3.3359
R-MTG	L-CER-8	129	−36	−57	−51	−3.4584
	L-LING	3542	3	−90	27	−4.4815
	L-SMA	775	−3	0	60	−4.3930
L-MTG	L-MOG	294	24	−81	15	−3.8720
R-MOG/ANG	L-MTP	732	0	−30	−33	−4.7765

*TAO, thyroid-associated ophthalmopathy; HC, healthy control; MNI, Montreal Neurological Institute; FC, functional connectivity; PostCG, postcentral gyrus; LING, lingual gyrus; CAL, calcarine; MTG, middle temporal gyrus; MOG, middle occipital gyrus; ANG, angular gyrus; CUN, cuneus; SOG, superior occipital gyrus; CER, cerebellum; SMA, supplementary motor area; MTP, middle temporal pole*.

### SVM Classification Results

**Figure 4:**
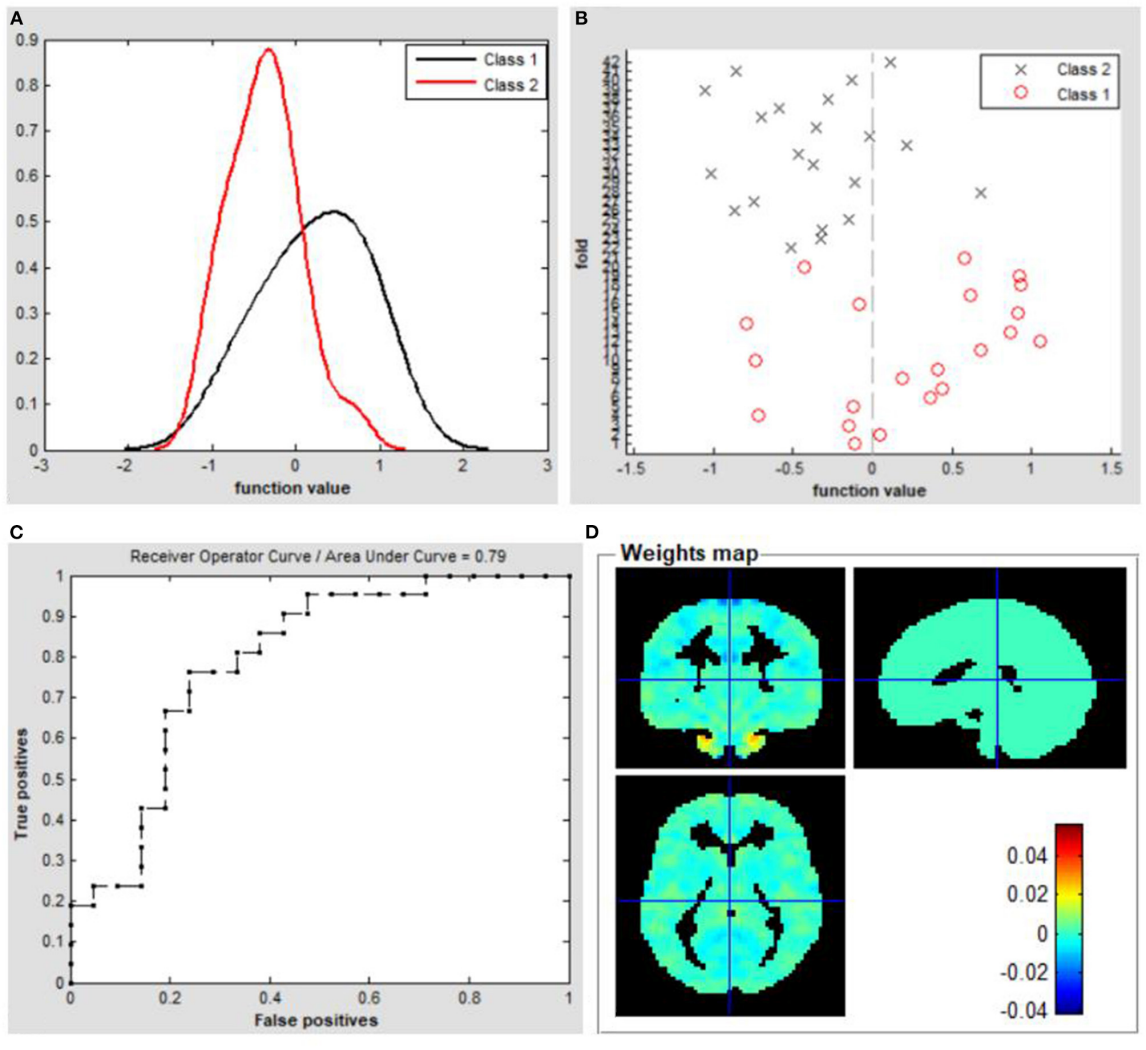
Classification results using machine learning analysis based on VMHC values. **(A)** Function values of two groups (class 1: TAO group; class 2: HC group); **(B)** Function values of two groups (class 1: TAO group; class 2: HC group); **(C)** The ROC curve of the SVM classifier with an AUC value of 0.79. **(D)** Weight maps for SVM models. TAO, thyroid-associated ophthalmopathy; HC, healthy control; VMHC, voxel-mirrored homotopic connectivity; ROC, receiver operating characteristic; SVM, Support vector machine; AUC, area under the curve.

To evaluate the classification ability of the SVM model, the accuracy, sensitivity, specificity and precision were calculated and the ROC curve of the classifier were illustrated in Figure 4. The performance of the classifier achieved an accuracy of 73.81% and AUC of 0. 79 for TAO vs HCs. Moreover, the SVM was applied to asses the classification ability of FC values (Supplementary Document).

## Discussion

To our knowledge, this is the first study to use the VMHC method combined with seed-based FC to explore interhemispheric functional coordination in patients with TAO and HCs. Compared with HCs, TAO patients showed significantly lower VMHC values in the bilateral PostCG/LING/CAL, MTG, and MOG/ANG. These reductions indicated extensive disruption of interhemispheric FC in patients with TAO. Furthermore, TAO patients exhibited decreased FC values between the right PostCG/LING/CAL and left LING/CUN/SOG, left PostCG/LING/CAL and right LING/MTG, right MTG and left CER-8/LING/MOG/ SMA, left MTG and right MOG, right MOG/ANG and left MTP (voxel-level *p* < 0.01, GRF correction, cluster-level *p* < 0.05). The performance of the SVM classifier achieved an accuracy of 73.81% and AUC of 0.79 for TAO vs HCs.

The occipital lobe contains most anatomical areas of the visual cortex, including the three Brodmann areas (Flores, 2002; Teng et al., 2018; Qi et al., 2021) associated with vision. The occipital lobe is associated with visual information processing; it has a critical role in the perception of facial emotion (Flores, 2002; Teng et al., 2018). BA19, the MOG, is regarded as a component of the dorsal visual stream, which has an important role in visual spatial processing (Tu et al., 2013; Chen et al., 2019a). The CAL is the most reliable anatomic landmark of the medial part of the occipital lobe; it contains the primary visual area. Lesions of the CAL are reportedly associated with visual field defects and the perception of phosphenes (Zhang et al., 2018; El Mohamad et al., 2019). The LING, located in the second visual area, receives strong feedforward connections from the CAL; the LING is also a crucial component of the dorsal visual pathway for visual processing and spatial memory (Klaver et al., 2015). The CUN is a wedge-shaped cortical region located inside the occipital lobe, posterior to the parietal occipital fissure and superior to the CAL. The CUN consists of both striatal and extrastriatal visual cortex; it receives direct input from the lateral geniculate body (Leopold, 2012; Meylakh and Henderson, 2022). Chen et al. (2021a) reported that patients with active TAO exhibited significantly decreased spontaneous brain activity in the MOG; this decreased activity was associated with visual deficits. Additionally, Tu et al. (2020) found abnormal FC density between the left precuneus and occipital gyrus in patients with dysthyroid optic neuropathy. The research revealed that dynamic abnormalities in thyroid hormone levels can lead to structural and functional abnormalities in the visual cortex. There is increasing neuroimaging evidence that patients with impaired vision have abnormal brain activity within the occipital cortex (Liang et al., 2017; Huang et al., 2019; Qi et al., 2021). Consistent with these findings, our study found that patients with TAO had significantly decreased interhemispheric FC in the MOG, LING, CAL, SOG and CUN; these results suggested the presence of disrupted interhemispheric functional synchronization in the visual network of TAO cohort, which corresponded to impaired visual function.

In addition to VMHC alterations in the visual brain network, we found that interhemispheric FC in the sensorimotor network was lower in patients with TAO than in HCs. The PostCG, a region in the parietal lobe, is the location of the primary somatosensory cortex; it is responsible for sensory functions such as the encoding of touch and pain (Shao et al., 2018; Chen et al., 2019b). Moreover, the PostCG is considered a component of the dorsal visual pathway and is involved in modulating vision (Zhu et al., 2016). Patients with TAO were reported to show decreased degree centrality in the PostCG, which suggested an aberrant degree of its functional connectivity in the parietal lobe (Chen et al., 2021b). Moreover, patients with TAO had significant thinning of the gray matter sheet in the PostCG, which might offer the neuroanatomical basis for psychological and ocular disturbances in the TAO cohort (Wu et al., 2020). The supplementary motor complex consists of the SMA, supplementary eye field, and pre-SMA; these areas are crucial for linking cognition to action (Hupfeld et al., 2017; Ruan et al., 2018). The SMA is located in the dorsomedial frontal cortex; it is involved in several functions related to motor sequence processing, planning, execution and inhibition (Li et al., 2006; Cona et al., 2017). Furthermore, the SMA is crucial for sensorimotor integration involved in generating actions (Ruan et al., 2018). Notably, a recent fMRI study revealed that, during silent lipreading, brain activity in the SMA decreased when an additional visual stimulus was included (Plata Bello et al., 2019). Consistent with these findings, we observed that patients with TAO had decreased interhemispheric FC within the sensorimotor network, which suggests impaired sensorimotor function in these patients.

Another important result in our study was that patients with TAO showed reduced interhemispheric FC strength in brain areas associated with cognitive function. The CER is located in the posterior fossa; it has important roles in visuospatial processing and eye movement (Stoodley and Schmahmann, 2010). Recent researches have revealed a critical role for the CER in higher cognitive functions (Izawa et al., 2012; Tong et al., 2021). Here, we demonstrated that patients with TAO had abnormal spontaneous neuronal activity in the CER; this activity was associated with cognitive impairment (Qi et al., 2021). We also found that patients with TAO showed lower interhemispheric FC in the bilateral ANG, compared with HCs. The ANG, located immediately above the Wernicke area, is an important connection area at the back of the brain. Because of its position, the ANG is presumably critical for integrating information among multiple input modalities and brain networks (Seghier, 2013). There is increasing evidence that the ANG is involved in reading, comprehension, visuospatial attention, spatial cognition, and episodic memory (Wang et al., 2010; Rugg and King, 2018). Humphreys et al. (2021) proposed a unifying model to explain complicated cognitive functions in the ANG; they suggested that the ANG served as an integrative dynamic buffer to combine information from local regions and brain networks. In addition, the ANG is a constituent part of the default mode network (DMN), which has been associated with spontaneous activity in rest situations; it is also involved in the unconscious processing of working memory tasks and implicit memory (Zuber et al., 2021). Thus, decreased interhemispheric FC in the ANG and CER might contribute to diverse cognition deficits in patients with TAO.

The MTG and MTP, core components of the temporal lobe, receive visual information input from the occipital lobe; they may constitute the higher areas of visual processing (Vedaei et al., 2021). Neural activity in the temporal region is associated with semantic processing including memory, visual, verbal, and executive functioning (Kang et al., 2021). The MTG is a critical component of the task positive network and the DMN, which is associated with externally oriented attention (Hu et al., 2020). Patients with TAO commonly complain of cognitive changes and emotional regulation deficits during the active stage. Patients with TAO reportedly have a predisposition to anxiety and attention disorder (Matos-Santos et al., 2001; Tsatsoulis, 2006). In the present study, we found decreased interhemispheric interactions in the MTG of patients with TAO. Therefore, integrating the neuroimaging findings with the cortical functions and the clinical psychiatric basis of the disease, we speculated that TAO might cause the MTG impairment, resulting in dysfunctions of visual attention, and visual processing.

An understanding of visual pathway lesions and cognitive deficits in TAO is important for the diagnosis and interventional treatment of the disease; combined VMHC and SVM assessments of TAO are thus meaningful. At present, the clinical diagnosis of TAO mainly depends on specific symptoms and computed tomography findings, but some patients lack specific symptoms. Therefore, we adopted an objective approach, SVM, to obtain a more reliable diagnosis for TAO. We also generated an ROC curve to assess the classification efficiency of the SVM approach. The overall identification accuracy of the SVM classifier was 73.81%; the AUC was 0.79, based on the LOOCV technique. These findings strongly support the hypothesis that the VMHC index can serve as a classification feature for distinguishing between the two groups through the application of supervised ML. Because of its robust objectivity and sensitivity, ML combined with multiparametric neuroimaging data might provide physicians with a powerful diagnostic tool in the near future.

However, the research had some potential limitations that should be ameliorated in the following studies. The first one is the relatively small sample size, although the results were encouraging. Due to the small sample size in the study, the generalizability of the findings was uncertain. Secondly, our study should include both the active and inactive patients with TAO and collect detailed clinical information scores and cognitive scales, which help understanding the neurological mechanisms underlying visual and cognitive disorders in these patients. Thirdly, a 73.81% accurate classification was not high enough. And we will expand the sample size and combine various machine learning methods including random forest or deep neural network to improve the accuracy in the next study. Additionally, the effect of physiological noise when performing rs-fMRI scanning was not completely eliminated, which might interfere with BOLD signals. Therefore, a combination of structural MRI, electroencephalogram, diffusion tensor imaging and fMRI might make the findings more convincing.

## Conclusion

We found that patients with TAO showed significant dysfunctional interhemispheric FC in the visual network, sensorimotor network, and brain areas associated with cognitive function. VMHC variability has potential value for accurately and specifically distinguishing patients with TAO from HCs. These findings may provide novel insights into the neurological mechanisms that underlie visual and cognitive disorders in patients with TAO.

## Data Availability Statement

The raw data supporting the conclusions of this article will be made available by the authors, without undue reservation.

## Ethics Statement

The studies involving human participants were reviewed and approved by Ethics Committee of Jiangxi Provincial People's Hospital. The patients/participants provided their written informed consent to participate in this study. Written informed consent was obtained from the individual(s) for the publication of any potentially identifiable images or data included in this article.

## Author Contributions

C-XQ, ZW, and XH contributed to data collection, statistical analyses, and wrote the manuscript. All authors contributed to the article and approved the submitted version.

## Conflict of Interest

The authors declare that the research was conducted in the absence of any commercial or financial relationships that could be construed as a potential conflict of interest.

## Publisher's Note

All claims expressed in this article are solely those of the authors and do not necessarily represent those of their affiliated organizations, or those of the publisher, the editors and the reviewers. Any product that may be evaluated in this article, or claim that may be made by its manufacturer, is not guaranteed or endorsed by the publisher.
