# Supplementary material for: Reduction of Interhemispheric Homotopic Connectivity in Cognitive and Visual Information Processing Pathways in Patients With Thyroid-Associated Ophthalmopathy
Source: Front Hum Neurosci. 2022 Jun 30;16:882114. doi: 10.3389/fnhum.2022.882114 (PMC9295451; doi:10.3389/fnhum.2022.882114)
Supplement: Supplementary file 1 [file Data_Sheet_1.docx]

The result of SVM to the FC data for prediction


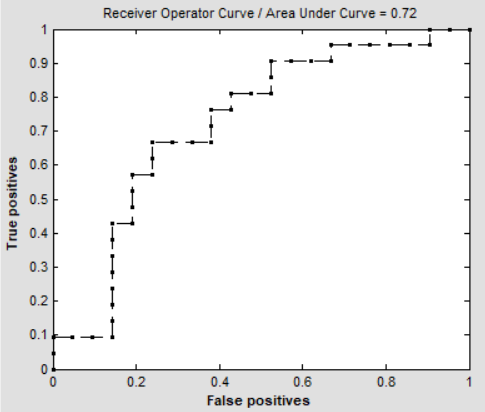


Figure1: FC of ROI1


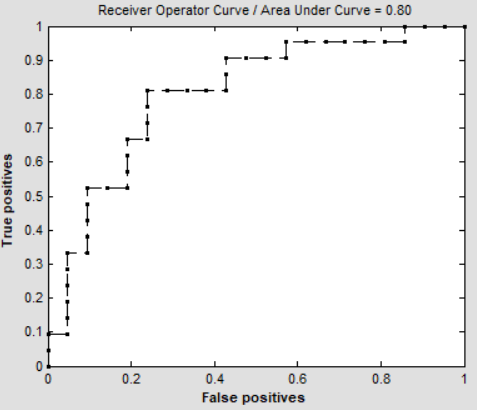


Figure2:FC of ROI2


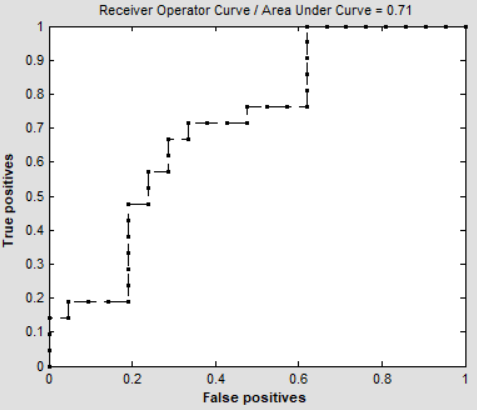


Figure3:FC of ROI3


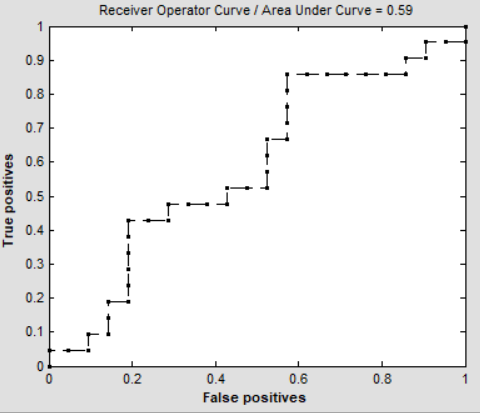


Figure4:FC of ROI4


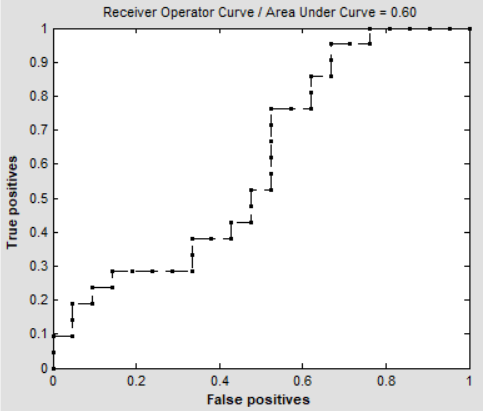


Figure5:FC of ROI5

Table1: The AUC of FC

| ROI1 | AUC=0.72 |
| --- | --- |
| ROI2 | AUC=0.80 |
| ROI3 | AUC=0.71 |
| ROI4 | AUC=0.59 |
| ROI5 | AUC=0.60 |
